# Systems biology to unravel Western diet-associated triggers in inflammatory bowel disease

**DOI:** 10.3389/fimmu.2025.1621334

**Published:** 2025-10-10

**Authors:** Špela Konjar, Evgen Benedik, Marko Šestan, Marc Veldhoen, Anže Županič

**Affiliations:** ^1^ Blood Transfusion Centre of Slovenia, Ljubljana, Slovenia; ^2^ Center for Proteomics, Faculty of Medicine, University of Rijeka, Rijeka, Croatia; ^3^ University Children’s Hospital, University Medical Centre Ljubljana, Ljubljana, Slovenia; ^4^ Biotechnical Faculty, University of Ljubljana, Ljubljana, Slovenia; ^5^ Department of Histology and Embryology, Faculty of Medicine, University of Rijeka, Rijeka, Croatia; ^6^ GIMM - Gulbenkian Institute for Molecular Medicine, Lisbon, Portugal; ^7^ Faculdade de Medicina da Universidade de Lisboa, Lisbon, Portugal; ^8^ Department of Biotechnology and Systems Biology, National Institute of Biology, Ljubljana, Slovenia

**Keywords:** IBD, western diet, immune cells, microbiota, systems biology

## Abstract

The global rise in inflammatory bowel disease (IBD) and other non-communicable diseases (NCDs) over the past five decades has coincided with the widespread adoption of a Western diet and lifestyle. These conditions, characterised by chronic inflammation, are shaped by complex interactions between genetic, environmental, immunological, and microbial factors. The Western diet rich in, refined sugars, unhealthy fats, ultra-processed foods and excess salt, is increasingly recognised as a major contributor to immune dysfunction, microbial dysbiosis, and compromised intestinal barrier integrity, all hallmarks of IBD. Systems biology offers a powerful framework for untangling the complexity of IBD by integrating large-scale biological data from various sources, leveraging computational modelling, high-throughput analyses, and network-based approaches to identify key regulatory pathways and molecular interactions driving disease progression. Complementary to this, nutritional epidemiology provides critical insights into the role of diet in IBD pathogenesis. By combining systems biology with nutritional epidemiology, researchers can move toward personalised dietary interventions and new therapeutic strategies, offering new opportunities for prevention and addressing the growing burden of IBD in societies adopting Western lifestyles. This review synthesise current findings and proposes integrated approaches for future precision prevention and treatment of IBD.

## 1 Introduction

**Figure 1:**
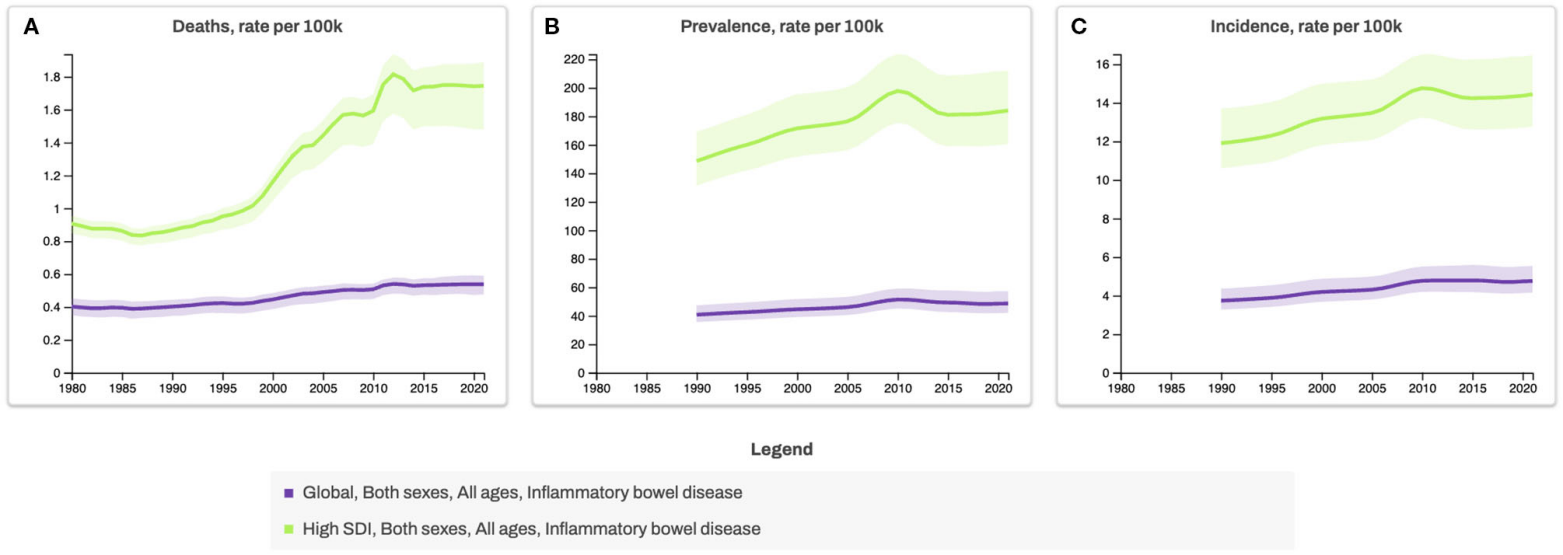
Global and High-Sociodemographic Index (SDI) Trends in Inflammatory Bowel Disease Mortality, Prevalence, and Incidence Rates, 1980–2020. Line graphs show age-standardized rates of **(A)** deaths, **(B)** prevalence, and **(C)** incidence of inflammatory bowel disease per 100,000 population for all ages and both sexes, stratified by SDI categories. The SDI is a composite measure from the Global Burden of Disease study that classifies countries by development level, based on income per capita, educational attainment, and fertility rate under age 25. The Global category represents the combined average across all countries, whereas High-SDI countries represent those with the highest development levels. *Data source for Figures 1, 2: Global Burden of Disease Collaborative Network. Global Burden of Disease Study 2021 (GBD 2021) Results. Seattle, United States: Institute for Health Metrics and Evaluation (IHME), 2022 (https://vizhub.healthdata.org/gbd-results, accessed 3 July 2025).

The global incidence of inflammatory bowel disease (IBD), which includes Crohn’s disease (CD) and ulcerative colitis (UC), has been rising steadily over the past few decades, particularly in regions undergoing rapid Westernisation (1) (**Figure 1**). UC typically presents as a continuous inflammation in the colon, beginning in the distal colon and advancing towards the proximal colon up to the cecum. This inflammation may result in the development of ulcers and bleeding. CD can manifest as patchy lesions anywhere in the gastrointestinal tract, often accompanied by inflammation, stenosis, and/or fistulas (2). IBD is characterised by alternating phases: periods of remission or quiescence, during which there is no intestinal inflammation and periods of active disease marked by the re-emergence of inflammation and related symptoms. The precise origins of IBD phenotypes and its extraintestinal manifestation remain incompletely understood (3–7). Notably, early-onset paediatric CD is associated with more severe disease progression and may be more strongly influenced by early environmental exposures, particularly dietary factors and microbiota composition (8, 9).

The prevalence of IBD is expected to rise to 1% in both developed and newly industrialised countries, mirroring the rise in Western-style dietary and lifestyle habits (10). With approximately 75% of patients experiencing persistent pattern of relapse this indicate the urgent need for a deeper understanding of these relapsing diseases (11–13).

**Figure 2:**
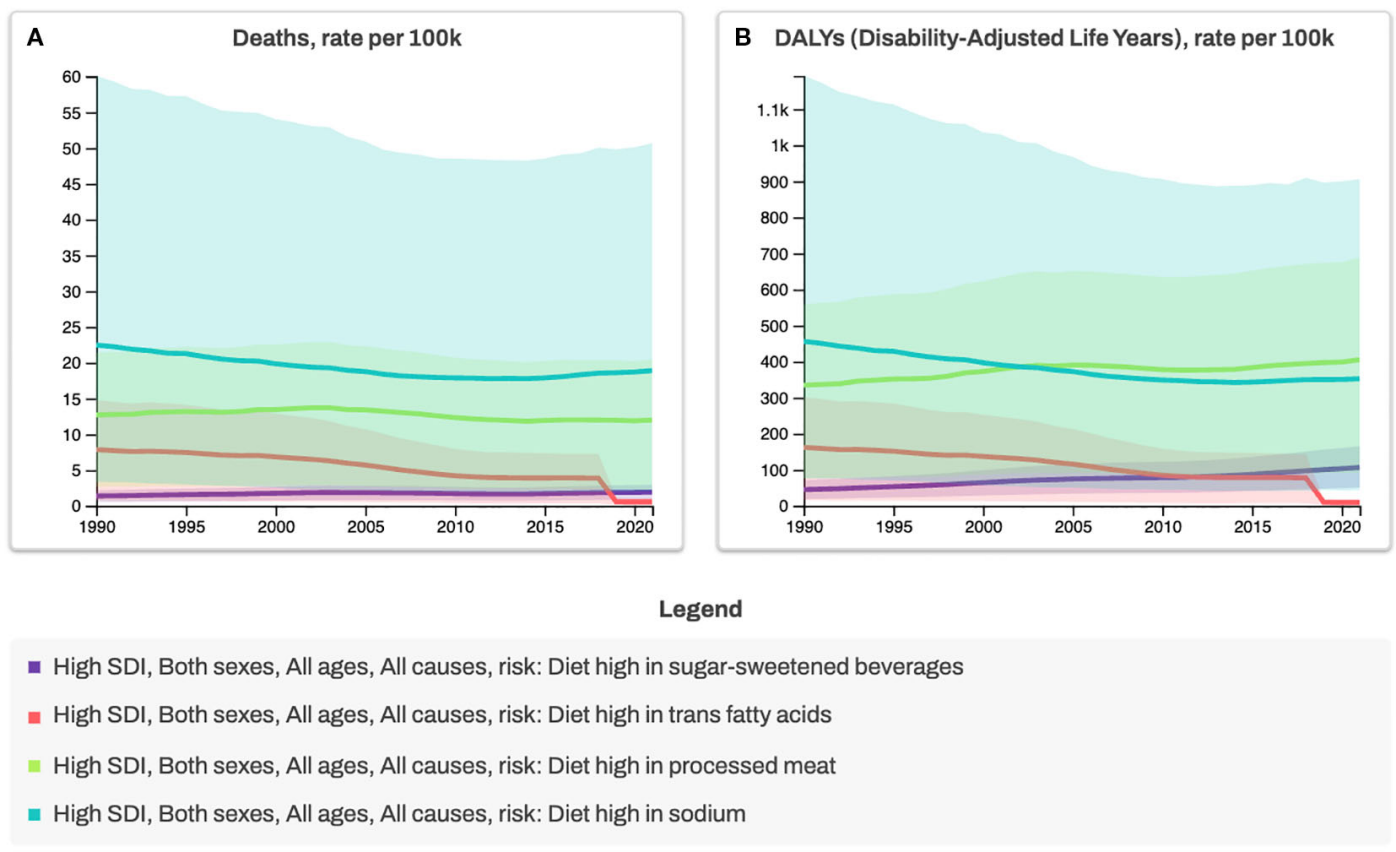
Trends in Mortality and Disability-Adjusted Life Years (DALYs) Attributable to Dietary Risk Factors in High-Sociodemographic Index (SDI) Countries, 1990–2020. Deaths **(A)** and diet-related DALYs **(B)** are shown as age-standardized rates per 100,000 population for all ages and both sexes. Analyses are stratified by four dietary risk factors: high intake of sugar-sweetened beverages, trans fatty acids, processed meat, and sodium. High- SDI countries represent nations with the highest income levels, greatest educational attainment, and lowest fertility rates, typically highly developed settings with advanced healthcare systems. Regulatory measures, such as trans fat bans (red line), illustrate the health benefits of reducing harmful dietary exposures and provide a model for comprehensive diet-related risk reduction strategies. *Data source for Figures 1, 2: Global Burden of Disease Collaborative Network. Global Burden of Disease Study 2021 (GBD 2021) Results. Seattle, United States: Institute for Health Metrics and Evaluation (IHME), 2022 (https://vizhub.healthdata.org/gbd-results, accessed 3 July 2025).

While genetic predisposition plays a role in disease susceptibility, genetics alone is unlikely to explain the rapid increase in cases. Instead, environmental factors, particularly diet and lifestyle changes, are thought to be major drivers of IBD onset and progression. This aligns with broader epidemiological trends showing a parallel rise in other non-communicable diseases (NCDs), such as obesity, cardiovascular disease, type 2 diabetes and types of cancer, all of which share chronic inflammation as a common pathological feature (1) (**Figures 1**, **2**). The Western diet, characterised by high consumption of refined carbohydrates, unhealthy fats, ultra-processed foods (UPFs), and excess salt, has emerged as a key contributor to systemic and gut-specific inflammation. Reinforcing the need to investigate dietary triggers in IBD pathogenesis.

**Figure 3:**
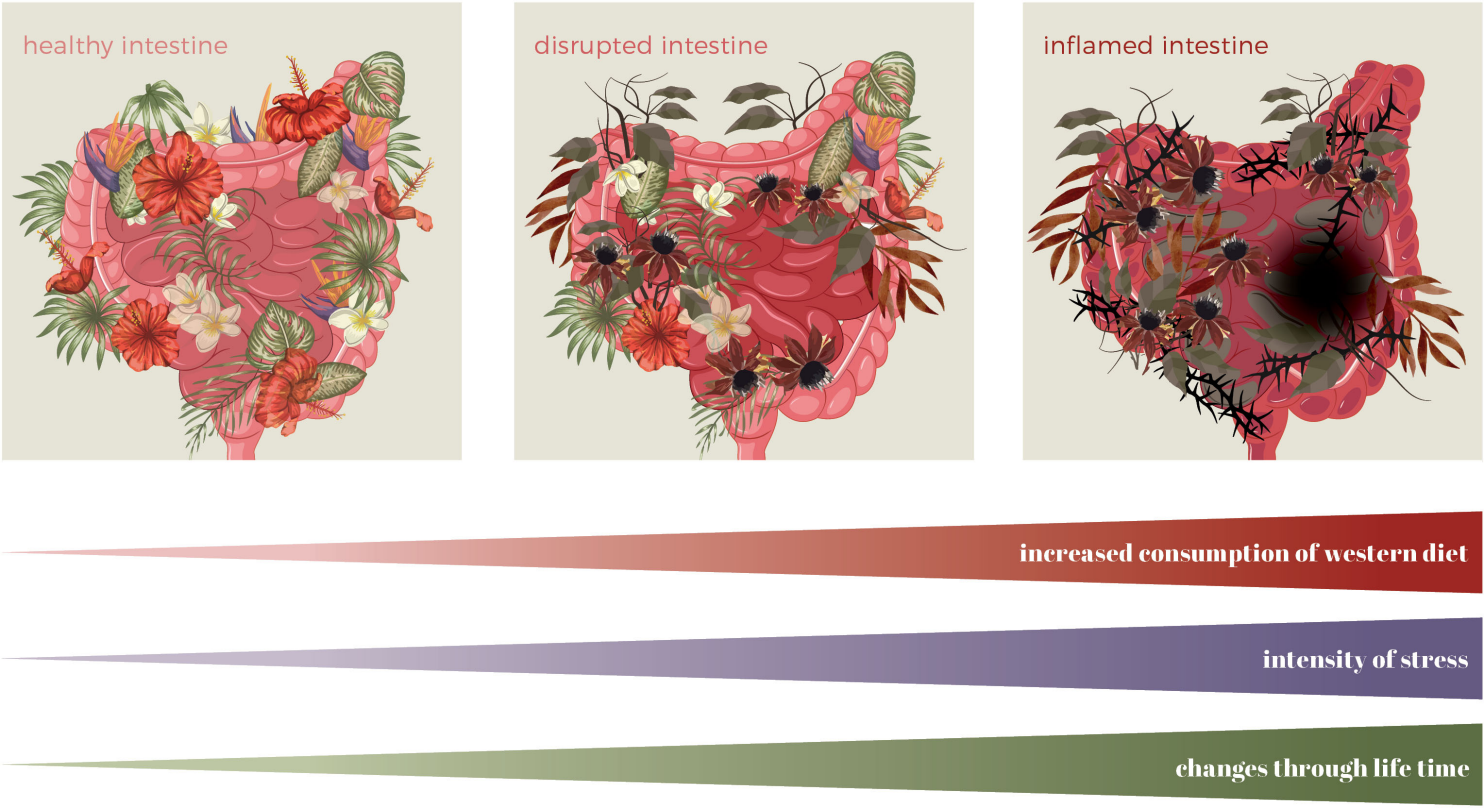
Changes in the intestine throughout a lifetime due to a Western diet and stress. Symbolic graphical presentation of intestinal landscape (microbiota=plants, intensity of inflammation=intensity of the colour of the intestine, lesion=black hole).

**Figure 4:**
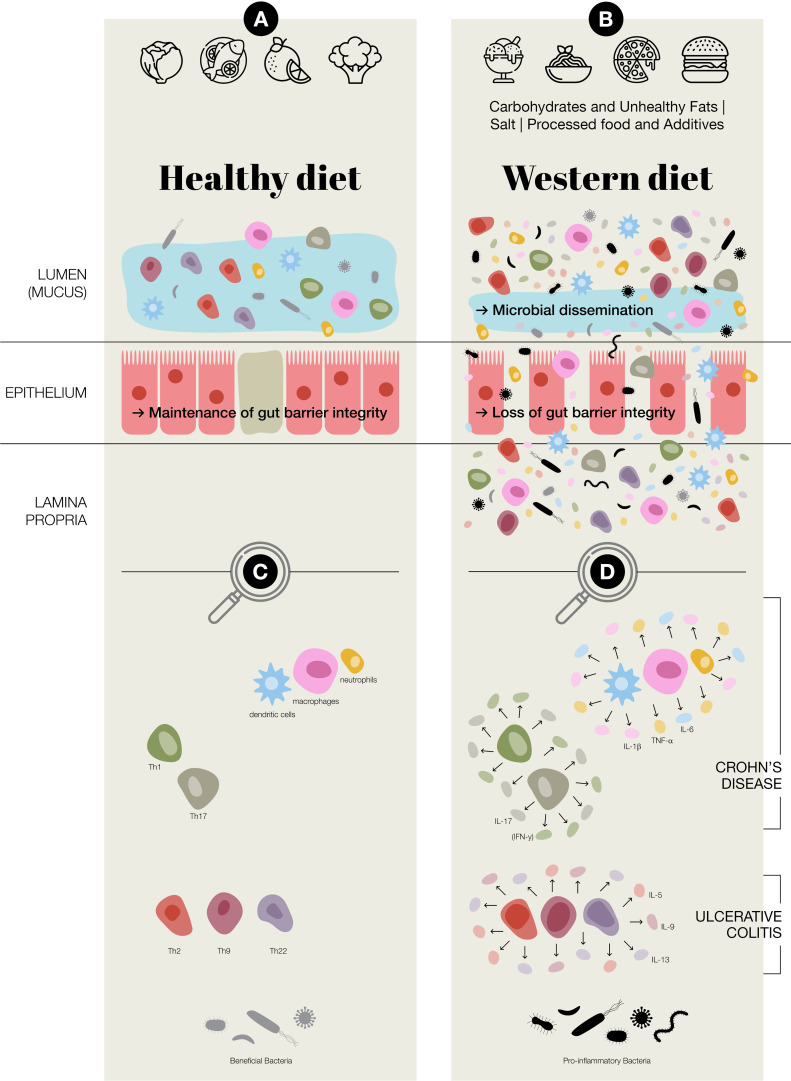
Potential of Western diet inducing chronic inflammation in the gut. **(A)** Healthy diet, maintenance of gut barrier integrity, normal immune regulation at the intestinal epithelial barrier, increased presence of beneficial bacteria; *Faecalibacterium prausnitzii* and *Bifidobacterium* **(B)** Western diet (Carbohydrates & Unhealthy Fats: alter gut microbiota, contribute to metabolic dysregulation and gut inflammation, increase intestinal permeability and endotoxemia. Processed food & Additives: disrupt gut microbial balance, erosion of the protective mucus layer, chronic low-grade inflammation. Salt: disrupting immune homeostasis, intestinal inflammation, alter the gut microbiota–immune system axis.); in general an aberrant activation of both innate and adaptive immune responses, loss of gut barrier integrity, a reduction in beneficial bacteria, such as *Faecalibacterium prausnitzii* and *Bifidobacterium*, and an increase in potentially pro-inflammatory species of bacteria, including adherent-invasive *Escherichia coli and Ruminococcus gnavus* **(C)** Predominate adaptive and innate immune cells present at intestinal epithelial barrier **(D)** Aberrant regulation/activation of predominate adaptive and innate immune cells in chronic inflammation in inflammatory bowel disease, which includes Crohn’s disease and ulcerative colitis.

A growing body of research suggests that diet influence IBD pathogenesis through multiple mechanisms, including alterations in gut microbiota composition (dysbiosis), increased intestinal permeability, immune dysregulation, and psychological disorders affecting the gut-brain axis (11, 14–16) (**Figures 3**, **4**). The gut microbiome plays a crucial role in maintaining intestinal homeostasis, influencing immune responses, and modulating inflammatory pathways. However, Western dietary patterns promote microbial imbalances that favour the increase of pro-inflammatory species while reducing beneficial bacteria. This shift in microbial composition is associated with a breakdown in gut barrier function, leading to increased permeability and facilitating the translocation of luminal antigens that can trigger mucosal inflammation (**Figure 4**). Despite accumulating evidence, pinpointing specific dietary triggers and their molecular effects remains challenging due to the complex interplay of genetic, microbial, and environmental factors. Traditional reductionist approaches to nutrition research, focusing on isolated nutrients or food components, often fail to capture these complexities. Instead, a systems-oriented perspective is needed to understand how diet influences gut health and disease risk at a broader level.

Systems biology provides a powerful framework for studying IBD by integrating multi-omic datasets and modelling host–microbiome–immune interactions (17, 18). While computational models hold promise for biomarker discovery and therapeutic target identification, many remain hypothetical and require experimental validation (18). The increasing availability of biological samples, including blood, stool, and intestinal biopsies, facilitates multi-dimensional profiling in IBD research (17, 19).

There are many unresolved questions regarding the relationship between IBD and the Western diet. In this review, we examine how components of the Western diet alter the intestinal landscape, contributing to immune dysfunction, microbial dysbiosis, and impaired intestinal permeability. We explore how systems biology and nutritional epidemiology offer complementary tools to unravel the complex diet–IBD interface, by integrating cutting-edge research methodologies, that could in the near future contribute to the development of more effective prevention and treatment strategies.

## 2 The gut microbiota-immune system axis in IBD

The interplay between the gut microbiota and the immune system is a fundamental aspect of human health, particularly in maintaining intestinal homeostasis, thereby preventing chronic inflammation. Microbes continuously stimulate the host’s immune system. Immune tolerance to components of the microbiota prevents risk of intestinal inflammation (20, 21). A fibre-rich diet can encourage the growth of beneficial gut bacteria, helping to maintain a balanced gut ecosystem. This healthy microbiota, in turn, supports a well-regulated immune response in the intestines—an important factor in managing IBD (21, 22). However, in individuals with a dysfunctional or hyperactive immune system, this tolerance is undermined, and can lead to chronic enteritis and colitis. IBD, is characterised by a disrupted relationship between host immunity and the microbial environment of the gut (21, 23, 24). Changes in microbial composition and metabolites caused by dysbiosis affect also autophagy, while impaired autophagy disrupts the mucosal barrier and immune balance, worsening gut inflammation (25, 26). The dynamic interactions between the gut microbiota, epithelial barrier, and immune system drive disease pathogenesis (**Figure 4**) (27). Understanding these complex interactions is crucial for developing novel therapeutic strategies aimed at restoring gut homeostasis and mitigating chronic inflammation.

### 2.1 Gut microbiota in IBD

The gut microbiota comprises of diverse communities of microorganisms, including bacteria, archaea, fungi, and viruses, which collectively perform essential functions in nutrient metabolism, immune modulation, and epithelial protection. In a healthy state, this microbial ecosystem is characterised by a balance between commensal and potentially pathogenic species, with dominant bacterial phyla including *Firmicutes, Bacteroidetes, Actinobacteria*, *and Proteobacteria*. These microorganisms contribute to gut health by fermenting dietary fibres into short-chain fatty acids (SCFAs), regulating mucosal immunity, and reinforcing the integrity of the epithelial barrier.

In IBD, however, a distinct shift in microbial composition, referred to as dysbiosis, has been consistently observed. This dysbiotic state is characterised by a reduction in beneficial bacteria, such as *Faecalibacterium prausnitzii* and *Bifidobacterium* (28), and an increase in potentially pro-inflammatory species, including adherent-invasive *Escherichia coli* and *Ruminococcus gnavus* (29). The loss of microbial diversity is particularly concerning as it is associated with impaired production of SCFAs, weakened mucosal protection, and heightened immune activation. Additionally, dysbiosis is not only a consequence but also a driver of disease progression, as an altered microbial landscape can influence immune cell differentiation and cytokine production, further promoting chronic inflammation (30, 31). Recent evidence suggests that dysbiosis may precede clinical disease onset, supporting its potential role as an early biomarker of IBD risk (17). Emerging evidence suggests that reduced microbial diversity and enrichment of pro-inflammatory taxa (e.g., *Enterobacteriaceae*) may serve as early predictors of IBD relapse (32).

Research in animal models and human studies has demonstrated a lasting imbalance in the gut microbiome in IBD (33–39). Reducing bacterial load with broad-spectrum antibiotics decreases the severity of colitis in IL-10 deficient mice (40). Another argument for the role of the microbiota is that CD lesions primarily occur in areas of the intestine with high bacterial populations such as ileum and colon (41).

#### 2.1.1 Microbiota-derived metabolites and their immunomodulatory effects during IBD

In addition to shaping immune responses via direct interactions with immune receptors, the gut microbiota exerts profound effects on host immunity through the production of microbial metabolites. Among these, SCFAs, the most well-studied being butyrate, acetate, and propionate, play a critical role in maintaining intestinal homeostasis. Butyrate, in particular, has been shown to enhance epithelial barrier function by upregulating tight junction proteins and stimulating mucus production (42). Furthermore, SCFAs exhibit anti-inflammatory properties by modulating the activity of macrophages, dendritic cells, and regulatory T cells (Treg), thereby promoting immune tolerance and suppressing excessive inflammation (43). Clinical studies further suggest that maintaining SCFA production, particularly butyrate, is associated with prolonged remission periods in IBD patients (34).

However, in the dysbiotic state observed in IBD, the production of beneficial microbial metabolites is often diminished, while harmful metabolites such as hydrogen sulphide and trimethylamine-N-oxide (TMAO) are increased (44). Hydrogen sulphide, although produced in small amounts by commensal bacteria under normal conditions, becomes cytotoxic at higher concentrations, impairing mitochondrial respiration, disrupting epithelial barrier integrity, and inducing oxidative stress in intestinal tissues (45). Elevated hydrogen sulphide levels can promote epithelial injury and sustain inflammatory signalling. Beyond activating NOD-like receptor family, pyrin domain containing 3 (NLRP3) inflammasome pathways, trimethylamine-N-oxide has been implicated in promoting mitochondrial dysfunction, enhancing oxidative stress, and impairing epithelial cell renewal, thereby exacerbating barrier breakdown and chronic mucosal inflammation (46). The accumulation of these harmful metabolites thus represents a key mechanistic link between dysbiosis and the perpetuation of intestinal inflammation in IBD (47).

#### 2.1.2 Microbiota and its impact on intestinal barrier dysfunction

Studies have shown that the microbiota of individuals with IBD is less functionally diverse, producing fewer SCFAs and more pro-inflammatory metabolites, which further exacerbate gut barrier disruption and immune dysregulation (48).

A key consequence of dysbiosis is its impact on the intestinal epithelial barrier, a complex structure that serves as the first line of defence against luminal pathogens and harmful antigens. The integrity of this barrier is maintained by tight junction proteins such as occluding, claudins and zonula occludens-1, which regulate paracellular permeability. In IBD, the Western diet has been implicated in disrupting tight junction assembly (49), leading to increased permeability and the phenomenon sometimes referred to as “leaky gut”, which is not a medical diagnosis. This reduction in barrier function facilitates the translocation of microbial-derived molecules, such as lipopolysaccharides and flagellin, which subsequently activate innate immune receptors and drive inflammatory cascades (50). The continuous exposure of the immune system to these microbial antigens can result in a state of hyperactivation, characterized by the excessive production of pro-inflammatory cytokines, including tumour necrosis factor-alpha (TNF-α) and interleukin (IL)-6, both of which are central to IBD pathogenesis (51). Additionally, oxidative stress caused by high-fat, low-fibre diets further compromise epithelial cell function, increasing susceptibility to inflammation and tissue damage (52, 53).

Emerging evidence suggests that dietary factors significantly influence barrier function, with Western dietary patterns rich in emulsifiers and artificial sweeteners further exacerbating epithelial dysfunction. Emulsifiers, such as polysorbate-80 and carboxymethylcellulose, have been shown to alter gut microbiota composition and promote low-grade inflammation, which can predispose individuals to barrier disruption (54). The increased permeability observed in IBD is also associated with alterations in the mucin layer, as reduced expression of mucin 2 (MUC2), a key mucin component, is frequently reported in patients with active disease (55).

### 2.2 Innate and adaptive immune responses in IBD

IBD is characterised by an aberrant activation of both innate and adaptive immune responses. The innate immune system, plays a crucial role in recognising and responding to microbial-associated molecular patterns through pathogen recognition receptors. In healthy individuals, innate immune responses are tightly regulated to prevent excessive inflammation. However, in IBD, innate immune cells, such as macrophages, dendritic cells, and neutrophils exhibit increased production of inflammatory mediators such as TNF-α, IL-1β, and IL-6 (56).

Neutrophils, in particular, are heavily recruited to the inflamed gut mucosa in IBD, where they release reactive oxygen species (ROS) and proteolytic enzymes that contribute to epithelial damage (57). Additionally, macrophages and dendritic cells in IBD patients display an altered cytokine profile, favouring the differentiation of pro-inflammatory T-helper (Th) cell subsets while suppressing regulatory mechanisms that could dampen inflammation (54, 58).

The adaptive immune system, which provides antigen-specific responses, also plays a central role in IBD pathogenesis. In CD, there is an overactivation of Th1 and Th17 cell immune responses, leading to excessive production of interferon-gamma (IFN-γ) and IL-17, both of which amplify inflammatory pathways and can drive tissue damage (59). Conversely, UC is primarily associated with a dysregulated Th2 cell response, characterised by increased secretion of IL-5 and IL-13, which disrupt epithelial barrier integrity and compromise the mucus layer (60).

Another key player in immune regulation is the population of Tregs, which are responsible for maintaining immune tolerance and suppressing excessive inflammation. In IBD, the balance between pro-inflammatory and regulatory immune responses is disrupted, with a reduction of Treg cells and an expansion of effector T cells. This imbalance results in persistent immune activation, prolonged tissue injury, and impaired mucosal healing. Importantly, the interaction between microbiota-derived metabolites and Treg cell differentiation and function is a growing area of research, with SCFAs playing a key role in promoting Treg expansion and function (61–64).

Significant challenges remain in elucidating the complex interplay within the gut microbiota-immune system axis during IBD. Key questions involve understanding precisely how and to what extent the homeostasis between the microbiota, the epithelial barrier, and the host immune system is disrupted (31, 47, 65–67). This includes determining the mechanisms driving the loss of microbial diversity and characterizing the functional consequences of altered microbial metabolite profiles during different disease stages (e.g., remission versus active disease). The inherent complexity arises from the multitude of interacting variables—spanning genetic, microbial, immune, and environmental factors—and their dynamic nature. Addressing these questions necessitates integrative approaches. As discussed further in Chapter 4, systems biology, which combines high-throughput ‘omics’ technologies with computational modelling, provides essential tools to dissect these interactions in both relevant animal models and human studies, paving the way for a more mechanistic understanding of IBD pathogenesis.

## 3 The role of diet in IBD

Inflammatory disorders are a common basis to NCDs and IBDs. NCDs include obesity, type 2 diabetes for which risk factor are carbohydrate and fats (68), different types of cancer for which ultra-processed food (UPF) is a risk factor (69) and cardiovascular diseases (CVD) for which a risk factor is salt (70). Given the epidemiological and mechanistic overlaps between IBD and NCDs, and the fact that these dietary risk factors are typical components of the Western diet, this chapter will explore how carbohydrates, fats, UPFs, and salt contribute to the development and exacerbation of IBD.

### 3.1 Carbohydrates and unhealthy fats as a risk factor for IBD

While the Western diet as a whole has been implicated in exacerbating IBD, individual dietary components exert specific effects on gut microbiota composition, immune responses, and epithelial integrity. Among the most concerning elements of this diet is the excessive consumption of refined carbohydrates, including sucrose and fructose, found in sugar-sweetened foods and beverages, and processed starches in energy-dense, nutrient-poor products, which contribute to metabolic dysregulation and gut inflammation (71). High-fructose corn syrup, a ubiquitous ingredient in processed foods and beverages, has been shown to increase gut permeability and oxidative stress, further aggravating inflammatory responses (71).

Carbohydrates include monosaccharides, disaccharides, oligosaccharides (typically comprising 3–10 units), and polysaccharides. Monosaccharides and disaccharides—often referred to as sugars—are generally soluble and are commonly consumed in the form of refined carbohydrates such as sucrose, fructose, and starch, found in energy-dense but nutrient-poor processed foods (72). Within the Western dietary pattern, refined sugars and processed grains (e.g. white flour and white bread) stand out for their detrimental impact on gut microbial composition and immune function (73).

The intake of refined sugars, particularly from sugar-sweetened beverages, has been shown to alter the gut microbiota, increasing the *Firmicutes/Bacteroidetes* ratio and reducing levels of beneficial butyrate-producing bacteria such as *Lachnobacterium* (74). These changes are linked to increased intestinal permeability and endotoxemia, which in turn promote local and systemic inflammation (75). Khan et al. investigated the effects of a high-sugar diet on colitis in rodent models and demonstrated that excessive intake of simple sugars exacerbated colitis in mice, whether administered before or after disease induction (76). Excessive sugar consumption has also been identified as a risk factor for obesity, type 2 diabetes, and cardiovascular disease (77). Furthermore, high sugar intake has been shown to induce physiological changes in the intestinal epithelium, including increased cell proliferation in crypts (78).

Fats, though an essential component of a balanced diet, can become harmful depending on their type and quantity. Unsaturated fats, often referred to as “healthy fats,” are associated with reduced risk of cardiovascular disease and metabolic syndrome (79, 80). In contrast, saturated and trans fats—prevalent in processed foods—are used to improve shelf life, texture, and flavours (81). These fats have been linked to intestinal dysfunction, including altered enteroendocrine cell activity, increased permeability, and mucosal inflammation (82). These effects contribute to chronic low-grade inflammation, a common feature underlying various NCDs (83).

Animal studies have demonstrated that high-fat diets significantly alter the gut microbiota, increasing the *Firmicutes/Bacteroidetes* ratio, reducing the antimicrobial function of Paneth cells, and elevating pro-inflammatory cytokines such as IFN-γ, TNF-α, IL-1β, and IL-6. These alterations promote bacterial translocation and systemic endotoxemia (72, 74).

The Western diet is often rich in fats associated with negative health outcomes, including refined seed oils (e.g. sunflower oil, palm oil), coconut oil, and processed foods high in omega-6 polyunsaturated fatty acids (84, 85). When consumed in excess, especially in the context of industrial food processing, these fatty acids may disrupt microbial homeostasis, promote the release of pro-inflammatory mediators, and compromise intestinal barrier integrity. Such alterations have been associated with an increased risk of IBD and colorectal cancer (86–90).

Likewise, diets rich in saturated and trans fats—particularly those derived from processed meats and fried foods—promote a microbial profile that favours pro-inflammatory species, thereby increasing susceptibility to intestinal inflammation (91). The consumption of red and processed meats has also been linked to the generation of harmful microbial metabolites, such as trimethylamine-N-oxide (TMAO), which contribute to both intestinal and systemic inflammation (92, 93).

### 3.2 Processed food and additives as a risk factor for IBD

The presence of food additives and emulsifiers in processed foods further complicates the dietary landscape of IBD. These substances may alter the composition and function of the gut microbiota, promoting the expansion of bacteria with pathogenic potential. In particular, dietary emulsifiers such as carboxymethylcellulose and polysorbate-80 have been shown to increase mucolytic bacterial activity, resulting in erosion of the protective mucus layer that separates the microbiota from the intestinal epithelium (53).

The thinning of this mucus barrier facilitates direct microbial–epithelial interactions, which can activate immune pathways and exacerbate inflammatory responses (66). Additionally, artificial sweeteners, widely used as sugar substitutes, have been implicated in metabolic dysregulation and shifts in microbial composition, potentially contributing to pro-inflammatory states within the gut (94).

Many food products within the Western diet are subjected to industrial processing techniques such as high-temperature cooking and extrusion. During these processing methods, harmful compounds such as advanced glycation end-products and other pro-inflammatory molecules can form through reactions between sugars, proteins, and lipids (95–97). Moreover, the Western diet is rich in UPFs, which typically contain a range of food additives, emulsifiers, preservatives, and synthetic colourings that may disrupt gut microbial balance and contribute to chronic low-grade inflammation (98). Synthetic food colorants, such as azo dyes (e.g. Red 40 and Yellow 6), are among the most commonly used additives in processed foods. Experimental evidence in genetically susceptible mouse models indicates that these compounds may induce colitis-like inflammation. Commensal bacteria, including *Bacteroides ovatus* and *Enterococcus faecalis*, have been shown to metabolize these dyes into 1-amino-2-naphthol-6-sulphonate sodium salt, a metabolite associated with colitis development (99).

Similarly, artificial sweeteners such as aspartame and sucralose have been shown to alter gut microbial composition, favouring the proliferation of pro-inflammatory bacterial species. Collectively, these dietary components can impair gut barrier integrity, enabling bacterial translocation and the passage of microbial antigens into the mucosal immune system, thereby exacerbating inflammation and mucosal damage (100).

However, there are challenges in interpreting data regarding the health effects of UPFs. The nutritional composition and quality of UPFs vary widely; for example, both seasoned canned tuna and ice cream are classified as UPFs despite their differing nutritional profiles (101). In a recent prospective cohort study, an association with CD risk was observed only when comparing extreme levels of UPF consumption (102). Furthermore, diets higher in UPF tend to also be high in fat and sugar and low in dietary fibre, making it difficult to determine whether health effects are due to ultra-processing itself or the overall nutritional composition. Although the precise mechanisms by which UPFs influence IBD risk are not yet fully understood, current clinical guidelines recommend that UPFs should be minimized in the diets of individuals with IBD or those at increased risk (103–105). Future studies employing systems biology approaches could help isolate the independent effects of food processing from overall nutrient composition.

### 3.3 Salt as a risk factor for IBD

Processed and preserved foods prevalent in the Western diet typically contain high levels of salt. However, the effects of this excessive salt intake on the human gut microbiota remain largely unknown (106). Several studies have linked UPFs to excessive salt consumption, which contributes to the development of hypertension and cardiovascular disease (107).

A high-salt diet (HSD) increased the abundance of *Bifidobacterium* and promoted greater gut permeability, which facilitated the intratumoural localization of *Bifidobacterium*. This, in turn, enhanced natural killer (NK) cell activity and contributed to tumour regression (108). Wilck et al. (2017) demonstrated that high-salt diets alter the gut microbiota–immune system axis by reducing populations of *Lactobacillus* spp., which may affect the production of SCFAs. This microbial shift was associated with enhanced activation of pro-inflammatory Th17 cells also limited metabolite availability cause cellular stress and enhance Th17 cell polarization (109, 110). Th17 cells play a pivotal role in IBD by producing pro-inflammatory cytokines such as IL-17A, IL-17F, and IL-22, which drive neutrophil recruitment, epithelial damage, and persistent inflammation (111). Excessive Th17 responses disrupt mucosal homeostasis, contributing to the chronic inflammatory milieu observed in CD and UC.

Similarly, Wu et al., 2013 showed that a modest increase in dietary salt concentration, promote IL-23 expression and enhance Th17 cell differentiation and pathogenic roles both *in vitro* and *in vivo* (112). An emerging body of evidence suggests that increased dietary salt intake, as a component of UPFs-rich diets, may contribute to the pathogenesis of IBD by disrupting immune homeostasis. Experimental models of colitis further support the role of high salt in exacerbating intestinal inflammation and promoting other immune-mediated disorders (113).

### 3.4 Components of diets that decrease symptoms of IBD

Conversely, some dietary components have been identified as protective factors in maintaining gut health and reducing IBD risk. Fibre-rich diets, promote the growth of beneficial gut bacteria and enhance the production of SCFAs, which are associated with reduced inflammation and improved gut barrier function (114).

Prebiotic fibres, such as inulin and resistant starch, selectively enhance the growth of commensal bacteria, leading to a more diverse and resilient microbiota. Unlike traditional diets, which support gut homeostasis through a diverse nutrient intake, the Western diet is reduced in key bioactive compounds such as dietary fibre and polyphenols (115).

The low intake of dietary fibre, has significant consequences for gut health, as fibre serves as a substrate for microbial fermentation. This process results in the generation of SCFAs—including butyrate, acetate, and propionate—which play a critical role in maintaining epithelial integrity, modulating immune responses, and supporting mucosal energy metabolism (116).

In addition to fibre, other bioactive dietary components such as polyphenols and omega-3 fatty acids have been recognized for their anti-inflammatory and immunomodulatory properties. Polyphenols, abundant in fruits, vegetables, and green tea, exert strong antioxidant effects and modulate inflammatory signalling pathways (117). Similarly, omega-3 fatty acids—commonly found in oily fish such as salmon and mackerel—have been shown to reduce the production of pro-inflammatory cytokines and enhance epithelial barrier function, offering potential therapeutic benefits for individuals with IBD (43).

The Western diet decreases microbiota diversity and consequently impact the composition of microbial metabolites. With metagenomics, metabolomics studies and development of models in systems biology the impact of diet and impact of individual component is already studied and will be extensively studied in the future (118). This will solidify which component of diet have predominate and harmful impact on intestinal microbiota and could be avoid in diets in the treatments of inflammatory disease such as NCDs and IBD.

## 4 Systems biology and nutritional epidemiology approaches in IBD and western diet

### 4.1 Systems biology in IBD research

High-throughput ‘omics’ technologies, including genomics, transcriptomics, proteomics, metabolomics, and metagenomics, has revolutionized IBD research by enabling integrated analyses of genetic, microbial, and immune dysregulation. Molecular profiling of patient samples has uncovered genetic risk loci, dysregulated pathways, metabolic alterations, and microbial imbalances, moving research beyond reductionist models. However, despite their promise, multi-omics-based systems approaches are rarely translated into clinical practice, primary due to challenges in data standardization, integration, and interpretation (18).

Genome-wide association studies (GWAS) represent a cornerstone of IBD genetics, identifying over 200 genetic loci linked to disease risk, particularly in pathways related to innate immunity, epithelial barrier function, and host-microbe interactions (17). While GWAS have been instrumental in mapping the genetic architecture of IBD, it’s important to note that the identified genetic predisposition explains only a portion of the overall disease variance, underscoring the significant contribution of environmental factors and gene-environment interactions.

Transcriptomic analyses, measuring gene expression levels, have provided critical insights into the molecular pathways perturbed in IBD. Studies analysing mucosal biopsies and circulating immune cells from IBD patients using bulk RNA sequencing have consistently revealed dysregulated pathways related to cytokine signalling, oxidative stress responses, and immune cell differentiation (19). More recently, the application of single-cell RNA sequencing (scRNA-seq) has added another layer of resolution. This powerful technique has allowed researchers to characterize the extensive heterogeneity of immune and stromal cell populations within the inflamed gut mucosa. Such studies have successfully uncovered distinct populations of pro-inflammatory cells, including specific subsets of Th17 cells, activated macrophages, and mucosa-associated invariant T (MAIT) cells, that are enriched or activated in patients with active IBD (19).

Metabolomics, the study of small molecule profiles in biological samples, complements transcriptomic and genomic data by revealing the functional metabolic outputs of the host and associated microbiota. Analyses of faecal, serum, and tissue metabolites from IBD patients have consistently identified significant alterations compared to healthy controls. Commonly observed changes include reduced levels of beneficial microbial-derived SCFAs like butyrate, alterations in bile acid profiles which impact fat digestion and signalling, and elevated markers indicative of increased oxidative stress within the intestinal environment (119). These metabolic shifts are often closely linked to the observed patterns of microbial dysbiosis and the compromised epithelial barrier function characteristic of IBD.

#### 4.1.1 Analysis focused on individual through time periods of remission and active state of disease

Understanding the multifactorial nature of IBD requires an integrative approach that captures the dynamic interplay between genetic, environmental, microbial, and immunological factors. Understanding the molecular dynamics during remission and active disease phases in individual IBD patients represents a crucial future research avenue. Monitoring time-resolved omics profiles could reveal unique disease trajectories and support personalized interventions. Particularly to understand the dynamics of change through these time points in composition of clusters of transcriptomes, metabolome etc. There are already papers where omics blood sample studies of IBD patient were able to predispose clinical relapse in IBD patient (120). The other publication identified biomarkers associated with functional remission in IBD (18, 121). These are some of the first studies that still need further replication and validation.

At the same it would be important to focus on specific individuals to characterize how different responses develop through times of remission and active disease. This could help to develop dynamic models that would be unique to the individual and, which may benefit in personalized precision medicine treatments. It was already shown that different mouse strains can immunologically respond differently to the same pathogen in the intestine. C57BL6/J and BALB/c mice differ in their tuft cell response to the protozoa *T. muris* but not the helminth *H. Polygyrus* in the *ileum* (122). There is a possibility that individuals develop different, potentially pathological, intestinal responses to the same stimuli or, that IBD pathogenesis develops similarly between groups of people. These differences and similarities could be detected and characterized with systems biology approaches.

#### 4.1.2 Specific versus complex, reductionist versus holistic

Omics data integration, network biology, and interactomes all reflect the inherent complexity, variability, and heterogeneity found in real-life physiological and pathological processes (123). Maintaining health amidst the constant challenges of growth, environmental influences, adaptation, nutrition, metabolism, immunity, behaviour, and both mental and physical demands require the precise coordination of countless physiological functions and their associated ‘omes´ (18, 124). The reductionist approach of seeking a single cause for complex diseases like IBD could be abandoned, as no single gene, environmental factor, microbial imbalance, or immune anomaly has been shown to cause typical CD or UC. Instead, a model that integrates all potential contributors offers a more effective path to understanding molecular mechanisms and identifying precise therapeutic targets (123). By integrating high-dimensional datasets systems biology provides a framework for identifying key molecular drivers and regulatory pathways involved in IBD pathogenesis (17).

With the three-predominant fronts of the intestinal landscape; microbiota, immune cells and epithelial the intestinal system is too complex to develop “clean” models. If we focus on a single omics method, analyses through time periods of remission and active disease and align the data with controls and develop through cluster the models with which we could gain useful, reliable and not too dispersed information. Initially the models can be developed in mice, which in comparison to studies in humans can be more interventional, and are environmentally and genetically controlled.

The understanding of IBD using appropriate models is not an easy reach. Such models, to be established and improved with reliable omics data, are difficult to develop due to the complexity of the multiple interactions, the dynamics of the disease evolution, the intricate anatomy, etc. Studying individual patients at several stages of disease and therapy using omics data is methodologically viable, incorporating sample collection in treatment protocols, documenting changes in medication, diet and lifestyle.

#### 4.1.3 Omics analysis before and after treatment of IBD

The advent of therapies targeting leukocyte trafficking (anti-integrins) or cytokines (anti-TNF, anti-IL-12/23) has transformed the management of IBD, significantly improving clinical remission and endoscopic healing while reducing disease-related morbidity (125). However, only a small subset of patients maintains long-term remission despite an initial response.

In patients with CD single-cell RNA-seq was applied to ileal CD lesions to address whether cellular heterogeneity contributes to TNFα antibody treatment resistance. They found that a subset of patients expressed a unique cellular module in inflamed tissues that consisted of IgG-producing plasma cells, inflammatory mononuclear phagocytes, activated T cells, and stromal cells, which they named the GIMATS module. This showed the existence of two qualitatively distinct subsets of disease, with distinct responses to anti-TNFα therapy (19). In a paper by Lee et al., 2021 (126) they found serum signatures of immune proteins reflecting microbial diversity identified patients more likely to achieve remission with anti-TNFα therapy.

The prediction of response to therapy in IBD patients based on results of multi-omics analyses appears to be at a preliminary stage and the results vary depending on the number and combination of the omics included in the prediction modelling. Perhaps more importantly, it still remains to be verified whether any of the newly described omics-based biomarkers are indeed better than traditional ones, such as C-reactive protein and fecal calprotectin (18, 127).

### 4.2 Systems biology methodologies for integration and modelling

While individual omics technologies provide valuable information about specific molecular layers, a deeper understanding of IBD requires integrating these diverse data types to capture the complex interplay between different biological processes. Beyond single-omics studies, systems biology methodologies aim to integrate multi-layered data to construct predictive models of disease mechanisms and therapeutic responses. Key approaches include statistical integration, network-based analysis, and computational modelling.

#### 4.2.1 Statistical multi-omics integration

This approach uses statistical methods to identify patterns, correlations, and associations across different omics datasets generated from the same samples or patient cohorts. The goal is often data-driven discovery of relationships without necessarily relying heavily on prior biological knowledge. Common techniques include:

- Multivariate statistics: Employing methods like Principal Component Analysis (PCA), Canonical Correlation Analysis (CCA), Partial Least Squares Discriminant Analysis (PLS-DA), and others to reduce data dimensionality and identify major axes of variation that span multiple omics layers (128). These can help distinguish between disease states or patient subgroups based on combined molecular profiles.
- Factor analysis methods: Tools like Multi-Omics Factor Analysis (MOFA+) aim to disentangle the sources of variation in multi-omics datasets, identifying latent factors that represent coordinated biological processes influencing multiple molecular layers simultaneously (129).

Examples in IBD research include studies integrating host transcriptomics with microbiome data to link gene expression patterns to microbial community structure (17) or combining multiple omics profiles from blood samples to identify signatures predictive of disease relapse (120).

#### 4.2.2 Network-based integration and disease maps

Network-based approaches leverage prior biological knowledge, typically stored in pathway databases (like KEGG, Reactome) or interaction databases (like STRING), to interpret multi-omics data in a biological context. This involves mapping omics data onto existing molecular interaction networks (e.g., protein-protein interaction networks, metabolic pathways, gene regulatory networks) to identify perturbed subnetworks or pathways.

A powerful extension of this concept is the Disease Maps initiative (130). Disease maps are community-driven efforts to build comprehensive, curated, and computationally accessible representations of the molecular mechanisms underlying specific diseases. They integrate information from literature and databases into standardized graphical representations (often using Systems Biology Graphical Notation - SBGN), providing a detailed overview of disease pathophysiology. These maps can be used as knowledge hubs and visualization tools, for instance allowing multiomics data to be overlaid and analysed in the context of known pathways and serve as a basis for computational modelling.

Platforms like MINERVA (131) provide the infrastructure to host, visualize, explore, and analyse these complex disease maps online. While dedicated, comprehensive IBD disease maps are still under development compared to fields like COVID-19 (132). The framework holds immense potential. An IBD map could integrate pathways related to immune cell signalling (TNF, IL-23/Th17, JAK-STAT), epithelial barrier regulation, host-microbe interactions, and the influence of genetic risk factors and environmental triggers like diet, providing a powerful tool for hypothesis generation and data interpretation.

#### 4.2.3 Computational modelling

Computational modelling aims to create mathematical or algorithmic representations of biological systems or subsystems to simulate their behaviour over time or under different conditions. These models often integrate data from multiple omics layers or are built upon the knowledge curated in network maps. Common modelling paradigms include:

- Ordinary Differential Equations (ODEs): Used to model the dynamics of molecular concentrations (e.g., cytokines, signalling intermediates) based on reaction rates, useful for simulating signalling pathways or metabolic fluxes.
- Agent-Based Models (ABMs): Simulate the behaviour and interactions of individual agents (e.g., immune cells, epithelial cells, bacteria) within a spatial context, useful for understanding tissue-level phenomena like immune cell infiltration or granuloma formation.
- Boolean (Logical) Models: Represent components (e.g., genes, proteins) as being either ON or OFF and use logical rules to describe interactions. These are particularly useful for modelling signaling networks derived from curated knowledge maps and predicting pathway activation states under perturbation, as demonstrated extensively in immunological contexts (133).

In the context of IBD, computational models offer promising tools to simulate the progression from intestinal homeostasis to inflammation, predict the effects of dietary changes on microbiota composition and subsequent immune responses, test therapeutic interventions in silico, and identify dynamic molecular patterns that could serve as early biomarkers for disease flares (18, 123). Although the development of complex, multi-scale models for IBD remains challenging due to the inherent heterogeneity of the disease and the dynamic interactions among genetic, microbial, immunological, and environmental factors (124), such approaches represent critical steps toward building predictive frameworks for understanding disease progression and personalizing therapy. Importantly, computational models primarily generate hypotheses that must be rigorously validated through experimental studies and clinical trials to ensure biological relevance and translational utility (16, 17).

### 4.3 Nutritional epidemiology and dietary interventions in IBD

Nutritional epidemiology helps identify dietary risk factors and informs prevention strategies. Randomised controlled trials (RCTs) in nutrition are limited by cost, compliance, and long-term adherence. Therefore, prospective cohort studies and case–control designs are essential for assessing associations between dietary patterns and disease outcomes.

Recent large-scale studies, such as the PURE cohort and the Nurses’ Health Study, show that high UPF intake correlates with increased IBD risk, especially CD. UPFs are rich in synthetic additives, refined sugars, and poor in fibre and micronutrients, all of which affect microbiota composition and immune regulation (98).

Conversely, adherence to anti-inflammatory dietary patterns, particularly the Mediterranean diet, is associated with improved outcomes. Rich in polyphenols, fibre, and unsaturated fats, the Mediterranean diet promotes microbial diversity, supports SCFA production, and reduces intestinal inflammation. Butyrate, the most abundant SCFA, exerts anti-inflammatory effects by promoting the differentiation of Tregs in the colon, inhibiting histone deacetylases, and suppressing pro-inflammatory cytokine expression, thereby strengthening mucosal immune tolerance (134). Additionally, specific therapeutic diets such as the specific carbohydrate diet, low fermentable oligo-, di-, monosaccharides and polyols diet, and CD exclusion diet are being explored for their utility in symptom management and disease modulation (135). Recent studies also suggest that partial enteral nutrition combined with targeted dietary interventions, such as an anti-inflammatory diet or a modified CD exclusion diet, can achieve comparable rates of clinical and endoscopic remission to exclusive enteral nutrition in paediatric CD, while offering improved tolerability (136, 137).

The convergence of systems biology with nutritional epidemiology enables the design of personalised dietary interventions tailored to an individual’s microbiome composition, immune profile, and metabolic status. Advances in machine learning and computational modelling further enhance the ability to predict individual responses to specific dietary interventions, paving the way for precision nutrition in IBD care (138). Pilot studies suggest that gut microbiota profiling can predict individual dietary responsiveness, enabling the development of targeted precision nutrition interventions for IBD patients (138, 139).

Emerging evidence highlights the critical importance of early-life nutrition in programming the gut microbiome and immune system. Breastfeeding, maternal fibre intake, and low exposure to emulsifiers during infancy are associated with enhanced microbial diversity and a lower risk of inflammatory diseases, including IBD later in life (140, 141). These findings underscore the need for preventive strategies beginning in early childhood.

## 5 Conclusions

Over the past several decades, the global rise in IBD has paralleled profound shifts towards Westernized dietary and lifestyle patterns. While genetic predisposition contributes to IBD susceptibility, growing evidence indicates that environmental triggers primarily diet, alterations in the microbiota, and immune dysregulation, play pivotal roles in disease onset and progression. These complexities necessitate interdisciplinary research strategies that move beyond reductionist models and embrace integrative approaches based on systems biology and nutritional epidemiology.

Systems biology has emerged as a transformative tool in IBD research, enabling the integration of multi-omic datasets to uncover novel disease mechanisms, identify biomarkers for early diagnosis, and support the development of precision medicine strategies. By combining genomic, transcriptomic, metabolomic, and microbiome data, researchers can construct predictive models that capture the dynamic interplay between host and environmental factors. These integrative models hold promise for improving patient stratification, optimising treatment strategies, and identifying novel therapeutic targets aimed at restoring gut homeostasis. However, successful clinical implementation of systems biology approaches requires overcoming key challenges, including the standardisation of data collection protocols, integration of heterogeneous datasets, and rigorous validation of computational predictions through experimental and clinical studies. In parallel, nutritional epidemiology has offered critical insights into the impact of long-term dietary exposures on IBD risk and disease progression. The association between Western dietary patterns, characterised by high consumption of UPFs, refined sugars, unhealthy fats, and food additives, and increased IBD incidence highlights the urgent need for dietary interventions as both a preventive and therapeutic strategies. In contrast, emerging evidence supports the protective effects of anti-inflammatory dietary patterns, such as the Mediterranean diet, which favourably modulate gut microbiota composition, enhance intestinal barrier integrity, and reduce systemic inflammation.

### 5.1 Future prospective

Looking forward, the convergence of systems biology with precision nutrition represents a transformative frontier in IBD research and clinical practice. Advances in machine learning and artificial intelligence will enable the development of personalised dietary recommendations based on an individual’s genetic profile, microbiome composition, and immune status. Future research should priorities elucidating the mechanistic links between specific dietary components, microbiota modulation, and immune responses in IBD pathogenesis. In parallel, the identification of reliable, clinically relevant biomarkers capable of predicting dietary responsiveness will be crucial for the personalization of nutritional interventions.

Another crucial area of investigation involves the impact of early-life nutritional exposures, including maternal diet, breastfeeding practices, and early childhood nutrition, on microbiota development, immune system programming, and long-term disease susceptibility. Given the ubiquity of food additives, emulsifiers, and artificial sweeteners in modern diets, their potential role in disrupting gut barrier function and promoting intestinal inflammation demands urgent and focused investigation. Although significant progress has been made in elucidating the complex interactions between diet, microbiota, and immune responses in IBD, numerous critical questions remain unanswered.

Future research must embrace interdisciplinary collaborations, combining expertise across gastroenterology, immunology, microbiology, bioinformatics, and nutritional sciences to develop comprehensive, patient-centred approaches. By leveraging the full potential of systems biology and nutritional epidemiology, the goal is to shift the paradigm of IBD care, from reactive disease management to proactive, personalized prevention, ultimately improving long-term outcomes and quality of life for individuals affected by this complex condition. Establishing causality remains a key challenge, and future progress will require large-scale, well-controlled, longitudinal studies to generate robust, evidence-based dietary guidelines for individuals at risk of, or living with, IBD.
